# Heavy Metal Removal by Bioaccumulation Using Genetically Engineered Microorganisms

**DOI:** 10.3389/fbioe.2018.00157

**Published:** 2018-10-29

**Authors:** Patrick Diep, Radhakrishnan Mahadevan, Alexander F. Yakunin

**Affiliations:** BioZone - Centre for Applied Biosciences and Bioengineering, Department of Chemical Engineering and Applied Chemistry, University of Toronto, Toronto, ON, Canada

**Keywords:** mining, heavy metal removal, bioaccumulation, bioremediation, bioextraction, genetic engineering, protein engineering, synthetic biology

## Abstract

Wastewater effluents from mines and metal refineries are often contaminated with heavy metal ions, so they pose hazards to human and environmental health. Conventional technologies to remove heavy metal ions are well-established, but the most popular methods have drawbacks: chemical precipitation generates sludge waste, and activated carbon and ion exchange resins are made from unsustainable non-renewable resources. Using microbial biomass as the platform for heavy metal ion removal is an alternative method. Specifically, bioaccumulation is a natural biological phenomenon where microorganisms use proteins to uptake and sequester metal ions in the intracellular space to utilize in cellular processes (e.g., enzyme catalysis, signaling, stabilizing charges on biomolecules). Recombinant expression of these import-storage systems in genetically engineered microorganisms allows for enhanced uptake and sequestration of heavy metal ions. This has been studied for over two decades for bioremediative applications, but successful translation to industrial-scale processes is virtually non-existent. Meanwhile, demands for metal resources are increasing while discovery rates to supply primary grade ores are not. This review re-thinks how bioaccumulation can be used and proposes that it can be developed for bioextractive applications—the removal *and recovery* of heavy metal ions for downstream purification and refining, rather than disposal. This review consolidates previously tested import-storage systems into a biochemical framework and highlights efforts to overcome obstacles that limit industrial feasibility, thereby identifying gaps in knowledge and potential avenues of research in bioaccumulation.

## Introduction

Mineral deposits are naturally occurring concentrations of chemical compounds in Earth's crust. Of importance are the metals in metallic mineral deposits that, once extracted and processed, are used as structural raw materials for infrastructure and as indispensable components of electronics and advanced materials for clean energy technology. At present, living standards are increasing in developing countries and the race to reduce carbon emissions to tackle climate change has become an international priority (Vidal et al., 2017). These large-scale human endeavors have gradually increased global demands for larger metal supplies, yet primary grade metal deposits are being discovered less frequently despite increased exploratory funding (Dunbar, 2017). In the mining and metal-refinery industries, cutting costs by de-prioritizing environmental stewardship has been common practice for a majority of the twentieth century as it allows for mining operations to be more economically feasible. However, this has led to a deterioration in public confidence given past and recent tailings dam failures (Azam and Li, 2010; Ali et al., 2017; Bowker and Chambers, 2017). Altogether, not only are there seemingly less primary grade deposits, but their accessibility is decreasing due to opposition by nearby residents and local governments that are safeguarded by larger organizations at the national and international level (Ali et al., 2017). Significant improvements have made the mining practice safer and more sustainable, which is paramount for re-building this trust and improving metal recovery. Many of these improvements include better wastewater effluents management and treatment.

Treating wastewater effluents laden with heavy metal ions (HMs) is challenging because it greatly depends on technoeconomic, environmental, and social considerations. This complexity precludes development of single technologies able to treat a multitude of wastewater effluents, so several technologies need to be deployed to curtail water pollution and remediate legacy sites in addition to their neighboring aquatic ecosystems (Akcil et al., 2015; Oyewo et al., 2018). Examples of presently used HM removal (HMR) technologies include: chemical precipitation, coagulants/ flocculants, membrane filtration, ion exchange, photocatalysis, and adsorption to inorganic materials. Advantages commonly associated with these conventional methods include rapid processing time, controllability, resilience to high concentrations of HMs, ease of operation, and well-understood molecular basis (Barakat, 2011; Fu and Wang, 2011; Gunatilake, 2015; Le and Nunes, 2016; Zhao et al., 2016; Carolin et al., 2017). These qualities satisfy many of the Eccles Design Criteria for HMR technologies that are important to consider because they ultimately determine the capital and operational costs (Eccles, 1995, 1999). However, a more modern set of design criteria are the Green Engineering Principles compiled by Anastas and Zimmerman (2003). While many of these conventional technologies can remove HMs extremely well, they may produce waste by-products that are difficult to dispose (Principle 2), and their energy requirements may be cost-prohibitive (Principle 3). More importantly, several are unsustainable because they utilize materials derived from non-renewable resources like coal and oil for activated carbon and ion exchange resins, respectively (Principle 12).

Biologically-driven HMR (bio-HMR) technologies use biomass to remove HMs from effluents and are often cited as cost-effective, environmentally friendly, and simple to operate. However, cost-benefit analyses, technoeconomic-environmental risk assessments, and industrial adoption are either poorly reported in literature or virtually non-existent. Whether bio-HMR satisfies Green Engineering Principles 2 or 3 is a matter of research and development. However, it does inherently satisfy Principle 12 because the biomass is often composed of waste products from the food and agriculture industry, or it is comprised of living and propagating cells that need nutrients derived from renewable resources. This reason alone calls for further exploration in bio-HMR as it has the potential to constitute a bulk of effluent treatment processes in the future where non-renewable resources will increase in price due to scarcity (Nyambuu and Semmler, 2014).

There are numerous biological phenomena that have been explored for their bio-HMR potential, but two have received notable attention: biosorption and bioaccumulation. These phenomena serve as “platforms” that can be manipulated for bio-HMR. Both are natural processes that all living cells undergo, so it is theoretically possible to screen all microorganisms and plants (dead or alive) for their bio-HMR potential. Moreover, the number of simulated and real wastewater effluent conditions that could be tested in these screens is countless (Gadd, 2009). Rather than exhaustively characterize combinations of species and conditions, other researchers have chosen to focus on a more direct and rational approach that leverages major developments in molecular biotechnology. This review first presents rationale for choosing bioaccumulative genetically engineered microorganisms (GEMs) as a bio-HMR technology (section Biosorption or Bioaccumulation?). Using a biochemical framework, this review then evaluates progress in developing GEMs that recombinantly express heterologous import-storage machinery (section Strategies and Limitations). To advance bioaccumulation, exploring its use for bioextraction is proposed by highlighting past efforts in developing bioprocesses and recent advances in molecular biotechnology (section Re-thinking Bioaccumulation).

## Biosorption or bioaccumulation?

Biosorption and bioaccumulation are not the same and should not be used interchangeably. Biosorption is the adsorption of particles to a biological matrix using physical interactions (electrostatic forces), chemical interactions (ion or proton displacement), complexation, or chelation. At neutral pH, the extracellular surface of microorganisms contains anionic moieties that provide binding sites for cationic HMs (Michalak et al., 2013; Fomina and Gadd, 2014). Numerous microbial species have been tested for their adsorption properties with HMs and are reviewed elsewhere (Volesky, 2007; Srivastava and Majumder, 2008; Vijayaraghavan and Yun, 2008; Wang and Chen, 2009; Ayangbenro and Babalola, 2017; Ilyas et al., 2017). Researchers have also engineered microorganisms to have recombinant metal-binding proteins and peptides on the extracellular surface, thus improving the capacity and specificity of these microbial biosorbents (Kuroda and Ueda, 2010, 2011; Ueda, 2016). This area has seen remarkable progress and has leveraged molecular biotechnology. However, since it is based on adsorption, it encounters challenges like those faced by some adsorption-based conventional methods, particularly the susceptibility to variations in pH and ionic strength that exists in heterogeneous wastewater effluents. Biosorbents also have limited lifespans because they often use dead biomass that degrades over time, and because fouling renders the binding sites unavailable (Gadd, 2009; Fomina and Gadd, 2014).

**Figure 1:**
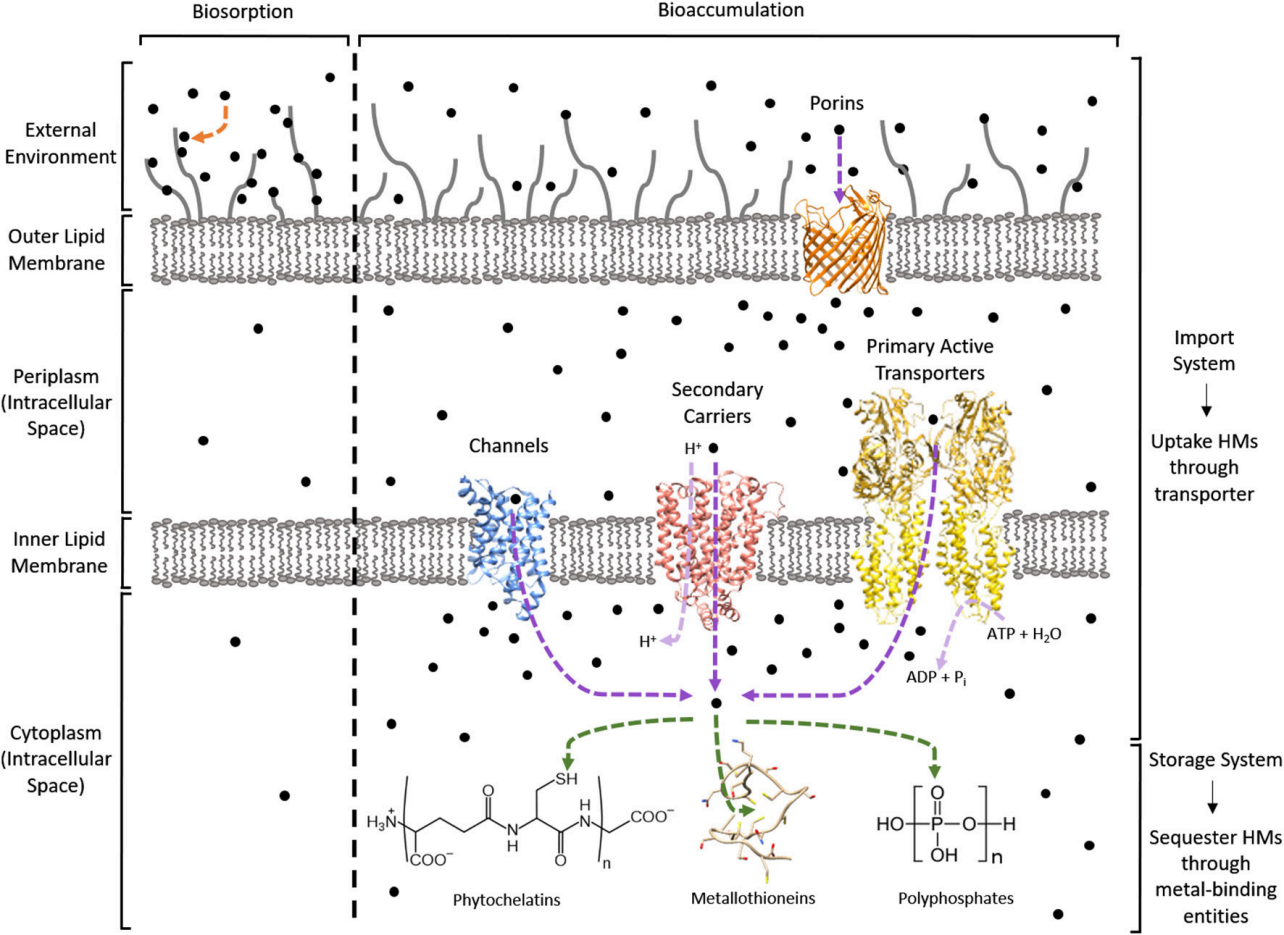
Bio-HMR technology overview using a Gram-negative bacterium. HMs are depicted as black circles. Biosorption is indicated by an orange arrow. Bioaccumulation can use an import-storage system where HMs are translocated across lipid membranes through transporters (purple arrows) into the cytoplasm for sequestration by metal-binding entities (green arrows). The light purple arrows specify the source of energy required for HM uptake: protons for secondary carriers and NTPs like ATP for primary active transporters. PDB structures used to visualize the protein machinery for these import-storage systems are 1LDA (blue), 4GBY (red), 3J09 (yellow), 2OMF (orange), and 1T2Y as the metallothionein.

In contrast, bioaccumulation is a metabolically-active process where microorganisms uptake HMs into their intracellular space using importer complexes that create a translocation pathway through the lipid bilayer (i.e., import system). Once inside the intracellular space, the HMs can be sequestered by proteins and peptide ligands (i.e., storage system; Malik, 2004; Mishra and Malik, 2013). This is visualized in Figure 1. The bioaccumulative capacity of a biomass for a target HM is a measure of performance commonly reported as μmol_x_ or mg_x_ per g_dry weight_, where x is the HM. These values are summarized in Table 1 for cadmium (Cd), cobalt (Co), copper (Cu), mercury (Hg), nickel (Ni), uranium (U), and the metalloid arsenic (As^3+^, As^4+^).

**Table 1:** Summary of bioaccumulation studies for various heavy metal ions.

**Heavy metal**	**Year of study**	Base chassis^*^	**Strategy (import or storage)**	**Analytical equipment, model**	Bioaccumulative capacity^**^(DW, dry weight)	**Time (h)**	**Starting concentration (mg_HM_/L)**	**References**
Nickel and Cobalt	2000	*Escherichia coli* JM109	Both	AAS, Perkin-Elmer 2380	0.88 mg_Ni_ g_DW_^−1^	1	0.58	Krishnaswamy and Wilson, 2000
	2003	*Escherichia coli* JM109	Both	AAS, Hitachi Z-8200	9.89 mg_Ni_ g_DW_^−1^	1	76	Deng et al., 2003
	2005	*Escherichia coli* SE5000	Both	AAS	7.05 mg_Ni_ g_DW_^−1^	1	75	Deng et al., 2005
	2008	*Escherichia coli* JM109, BL21-DE3, MC4100, ARY023	Import	Integral γ-counter coupled to a well type (2 × 2 in.) NaI Tl detector	0.012 mg_Co_ g_DW_^−1^	2	0.000002	Raghu et al., 2008
	2013	*Escherichia coli* JM109	Both	ICP-OES, Perkin-Elmer OPTIMA 7000	60 mg_Ni_ g_DW_^−1^	2	160	Deng et al., 2013
	2014	*Escherichia coli* K-12 MG1655	Import	ICP-MS; TriCarb 2100TR scintillation counter	4.8 mg_Co_ g_DW_^−1^, 6 mg_Ni_ g_DW_^−1^	0.17	2.9 (Co and Ni)	Duprey et al., 2014
	2015	*Deinococcus radiodurans* R1	Import	Integral γ-counter coupled to a well type (2 × 2 in.) NaI Tl detector	0.012 mg_Co_ g_DW_^−1^	1.5	0.0005	Gogada et al., 2015
Arsenic Species	2004	*Escherichia coli* BLR (DE3)	Storage	AAS, Shimadzu AA6701	0.17 ${\text{mg}}_{\text{As}}^{3+}$ g_DW_^−1^	48	0.75	Kostal et al., 2004
	2008	*Saccharomyces cerevisiae* BY4742, 15616	Storage	AAS, Perkin-Elmer	0.22 ${\text{mg}}_{\text{As}}^{3+}$ g_DW_^−1^	15.5	1.5	Singh et al., 2008a
	2008	*Escherichia coli* JM109	Both	not specified	0.61 ${\text{mg}}_{\text{As}}^{3+}$ g_DW_^−1^	0.33	0.000035	Singh et al., 2008b
	2009	*Escherichia coli* JM109	Storage	AAS, Beijing Jida Instrument AFS-820, Perkin-Elmer 600; ICP-OES, IRIS In- trepid II XSP	0.32 ${\text{mg}}_{\text{As}}^{3+}$ g_DW_^−1^	3	3.75	Su et al., 2009
	2010	*Escherichia coli* JM109	Both	AAS, Perkin-Elmer AAnalyst 800	1.25 ${\text{mg}}_{\text{As}}^{3+}$ g_DW_^−1^	3	0.75	Singh et al., 2010
	2010	*Saccharomyces cerevisiae* BY4742, 15616	Both	AAS, Perkin-Elmer AAnalyst 800, SIMAA 6000	0.28 ${\text{mg}}_{\text{As}}^{3+}$ g_DW_^−1^	10	7.5	Shah et al., 2010
	2012	*Saccharomyces cerevisiae* BY4742, 15616	Import	AAS, Perkin-Elmer	0.74 ${\text{mg}}_{\text{As}}^{4+}$ g_DW_^−1^	1	0.012	Shen et al., 2012
	2013	*Escherichia coli* BL21 (DE3)	Storage	AAS, Shanghai Spectrum Instruments SP-3802AAPC	5.24 mg_MMA_ g_DW_^−1^ 3.92 mg_DNA_ g_DW_^−1^	1	1000 (MMA and DMA)^***^	Yang et al., 2013
	2014	*Corynebacterium glutamicum* 13032	Both	ICP-MS, Varian	2.16 mgAs^4+^ g_DW_^−1^	2	7.5	Villadangos et al., 2014
Cadmium	1995	*Escherichia coli* TB1	Storage	Liquid scintillation counter, Packard	1.91 mg_Cd_ g_DW_^−1^	1	1.12	Pazirandeh et al., 1995
	1999	*Escherichia coli* XL1-blue	Import	AAS, Perkin-Elmer 2380	0.068 mg_Cd_ g_DW_^−1^	0.17	2.24	Hao et al., 1999
	2002	*Escherichia coli* JM109	Storage	AAS, Shimadzu AA646	10.5 mg_Cd_ g_DW_^−1^	96	56.21	Yoshida et al., 2002
	2003	*Mesorhizobium huakuii* subsp. Rengei B3	Storage	AAS, SAS7500A	4.047 mg_Cd_ g_DW_^−1^	40	3.37	Sriprang et al., 2003
	2007	*Escherichia coli* JM109	Both	AAS, Hitachi Z-8200	63.26 mg_Cd_ g_DW_^−1^	1	60	Deng et al., 2007
	2007	*Escherichia coli* JM109	Both	AAS, Perkin-Elmer AAnalyst 800	3.55 mg_Cd_ g_DW_^−1^	/	2.24	Kang et al., 2007
	2013	*Escherichia coli* BL21, MG1655	Both	AAS, Shimadzu AA6501	7.5 mg_Cd_ g_DW_^−1^	5	56.21	Chang and Shu, 2013
	2015	*Escherichia coli* BL21	Storage	AAS Hitachi Z-2000	~6 mg_Cd_ g_DW_^−1^	2	56.21	Gong et al., 2015
Copper	2003	*Escherichia coli* BL21	Storage	AAS, Varian 220Z	145 mg_Cu_ g_DW_^−1^	6	0.01	Ueki et al., 2003
	2017	*Saccharomyces cerevisiae* BY4743	Storage	ICP-AES, Varian	103.3 mg_Cu_ g_DW_^−1^	120	330	Geva et al., 2016
Mercurial Species	1997	*Escherichia coli* JM109	Both	AAS	~1.60 mg_Hg_ g_DW_^−1^	1	1	Wilson, 1997
	1997	*Escherichia coli* JM109	Both	ICP-AES	17.65 mg_Hg_ g_DW_^−1^	1	26	Chen and Wilson, 1997
	2001	*Escherichia coli* JM109	Both	AAS, Coleman Model 5B	3.81 mg_Hg_ g_DW_^−1^	1	1	Bae et al., 2001
	2002	*Escherichia coli* XL1-blue	Both	AAS, Nippon Instruments	1.07 mg_Hg_ g_DW_^−1^ 1.48 mg_C6H5Hg_ g_DW_^−1^	0.17	3.21	Pan-Hou et al., 2002
	2003	*Escherichia coli* XL1-blue	Both	AAS	178.72 mg_Hg_ g_DW_^−1^ 215.5 mg_C6H5Hg_ g_DW_^−1^	72	2 (Hg and C_6_H_5_Hg)	Kiyono et al., 2003
	2004	*Escherichia coli J*M109	Both	AAS	6.86 mg_Hg_ g_DW_^−1^	25	3.65	Zhao et al., 2004
	2006	*Escherichia coli* JM109	Both	AAS	25 mg_Hg_ g_DW_^−1^	14	4	Deng et al., 2006
	2011	*Rhodopseudomonas palustris* GIM1.167	Both	ICP-OES, Perkin-Elmer OPTIMA 7000	77.58 mg_Hg_ g_DW_^−1^	2	90	Deng and Jia, 2011
	2018	*Escherichia coli*	Storage	AAS	4.012 mg_Hg_ g_DW_^−1^	6	6	Shahpiri and Mohammadzadeh, 2018
Uranium	1998	*Escherichia coli* DH5α	Storage	Modified spectrophotometric method using fluorophore	151 mg_U_ g_DW_^−1^	24	238	Basnakova et al., 1998
	2013	*Deinococcus radiodurans* R1	Storage	Arsenzo (III) Reagent	10700 mg_U_ g_DW_^−1^	1	2380	Kulkarni et al., 2013
Multi Metal	2002	*Mesorhizobium huakuii* subsp. Rengei B3	Storage	AAS, SAS7500A	0.71 mg_Cd_ g_DW_^−1^ 0.16 mg_Cu_ g_DW_^−1^	2 mos	22.5 (Cd), 15.9 (Cu)	Sriprang et al., 2002
	2003	*Escherichia coli* M15REP4	Storage	ICP-OES, Varian	0.81 mg_Cd_ g_DW_^−1^ 0.17 mg_Cu_ g_DW_^−1^	3	2.25 (Cd), 1.27 (Cu)	Sauge-merle et al., 2003
	2004	*Escherichia coli* JM109	Import	AAS	0.96 mg_Cd_ g_DW_^−1^ 0.29 mg_Cu_ g_DW_^−1^	0.5	2.25 (Cd), 1.27 (Cu)	Zagorski and Wilson, 2004
	2005	*Saccharomyces cerevisiae* DTY165, DTY167	Storage	ICP-AES, Perkin-Elmer Optima 4300DV	0.28 mg_Cd_ g_DW_^−1^ 0.10 ${\text{mg}}_{\text{As}}^{3+}$ g_DW_^−1^	24	0.75 (As^3+^), 2.25 (Cd)	Young et al., 2005
	2006	*Escherichia coli* DH5α	Storage	AAS, GBC Scientific Equipment Model 932+	52.5 mg_Pb_ g_DW_^−1^ 23.8 mg_Cu_ g_DW_^−1^ 14.5 mg_Cd_ g_DW_^−1^	24	300 (Cd, Cu, and Pb)	Kao et al., 2006
	2011	*Escherichia coli* BL21 (DE3)	Storage	AAS, Hewlett Packard	6.36 mg_Cd_ g_DW_^−1^ 7.59 ${\text{mg}}_{\text{As}}^{3+}$g_DW_^−1^	4	7.5 (As^4+^) and 22.5 (Cd)	Ma et al., 2011
	2012	*Escherichia coli* TB1, BL21(DE3), LF20012	Storage	ICP-AES, Varian	1.51 mg_Cd_ g_DW_^−1^ 0.49 ${\text{mg}}_{\text{As}}^{3+}$ g_DW_^−1^ 0.31 mg_Cu_ g_DW_^−1^ 0.94 mg_Hg_ g_DW_^−1^ 1.79 mg_Pb_ g_DW_^−1^	3	1.5 (As^3+^), 2.25 (Cd), 1.27 (Cu), 1 (Hg), 4.14 (Pb)	Sauge-Merle et al., 2012
	2014	*Escherichia coli* BL21 (DE3)	Storage	ICP-AES, Perkin-Elmer Optima 7300DV	0.13 mg_Cd_ g_DW_^−1^ 0.057 mg_Cu_ g_DW_^−1^	6	33.7 (Cd), 19.1 (Cu)	He et al., 2014
	2015	*Escherichia coli* Rosetta (DE3)	Storage	ICP-MS, Varian	2.24 mg_Cd_ g_DW_^−1^ 12.39 mg_Cu_ g_DW_^−1^ 0.82 mg_Hg_ g_DW_^−1^	12	168 (Cd), 159 (Cu), 20 (Hg)	Li et al., 2015

* *Base Chassis refers to the strain that was directly used in bioaccumulation studies or was mutated for a specific purpose and later used in bioaccumulation studies. These subsequent mutants containing knock-outs are not included*.

** *Bioaccumulative capacity values are adapted from their respective publications for general comparison and should not be used for quantitative analysis. Some values were estimated from figures*.

*** *MMA, monomethylarsonic acid; DMA, dimethylarsinic acid*.

“Metabolically-active” implies bioaccumulation requires the host cell to be alive, which imposes unique challenges: nutrient feeds for sustaining and propagating biomass, level of aeration to accommodate aerobic/anaerobic needs, and accidental release of GEMs into the environment. It also implies the process involves proteins in the cytosol and those embedded in the lipid membrane. This imposes more unique challenges: decreased cell viability due to the expression of heterologous import-storage proteins that are not mandatory for growth, excessive protein aggregation, and loss-of-phenotype due to competition by native microorganisms.

### Wildtype or engineered microorganisms?

Both biosorption and bioaccumulation have challenges that limit their use for HMR, but they arise differently and require distinct approaches to address them (Chojnacka, 2010; García-García et al., 2016; Hansda et al., 2016). Solutions for biosorption are often inspired from solutions for conventional sorption-based methods: extensive screening of microbial species with attractive adsorption properties, chemical modification of the anionic moieties on the outer surface, and the genetic engineering of the extracellular space to have metal-binding proteins and peptides (Rangabhashiyam et al., 2014; Ueda, 2016; Ayangbenro and Babalola, 2017).

Conversely, limitations of bioaccumulation concern the inner architecture of the cell: the gene and protein expression levels, and the stress response to the often toxic composition of wastewater effluents. While the former necessitates synthetic biology to optimize trade-offs between recombinantly expressing protein machinery (i.e., import-storage system) and cellular growth, the latter poses an important question: how do wildtype microorganisms compare with GEMs for treating wastewater effluent through bioaccumulation? Mishra and Malik (2013) provide the most recent summary of wildtype yeast, fungi, bacteria, and algae with bioaccumulative capacities larger than those of GEMs reported in Table 1 of this review. However, these wildtype microorganisms were mostly incubated in simulated and real wastewater effluents for longer periods, which may explain the differences. While it is valuable to compare which wildtype microorganism or GEM can treat an effluent sample better and faster, none outstandingly outperform others as shown by the paucity of industrial adoption of bio-HMR.

It may be more valuable to compare practical implications than performance. Using wildtype microorganisms adapted to a wastewater effluent's nutrient composition confers a survival advantage over non-native GEMs that carry the burden of expressing an import-storage system that it is not mandatory for growth. Although true, wildtype microorganisms are less robust than GEMs because the protein machinery they use to uptake and sequester HMs are transcriptionally and translationally controlled by genetic regulatory systems that the species has evolved. It is unlikely that wildtype microorganisms will bioaccumulate HMs beyond their minimum inhibitory concentrations, thus imposing a limit to their bioaccumulative performance. GEMs use characterized genetic regulatory elements (e.g., promoters, ribosome binding sites, terminators) chosen by the user for induction with external stimuli that they can manipulate in batch and continuous bioprocesses (section Scale-up). Therefore, GEMs may offer superior control over when, how long, and how strongly an import-storage system is expressed to allow for predictable uptake and sequestration of HMs. Transferring the gene sequence encoding this import-storage system to microorganisms that robustly grow in single or multiple wastewater effluents may allow for targeted and generalized bio-HMR through bioaccumulation (section Chassis Selection). This is also tied to GEMs being more versatile because the import-storage systems are comprised of proteins that have usually been biochemically studied, which enables protein engineering for modification of the HM specificity and selectivity (section “Omics” and Protein Design).

This review does not suggest there cannot be synergies between biosorption and bioaccumulation by wild-type microorganisms with bioaccumulation by GEMs. However, it does argue that the specificity and selectivity conferred by import systems, and capacity afforded through storage systems can be engineered to create GEMs that are potentially more robust and versatile. By first understanding the state of research in bioaccumulative GEMs, gaps in knowledge can be identified and unexplored potential avenues can be delineated to direct future research to accomplish these goals.

## Strategies and limitations

To genetically engineer microorganisms for enhanced bioaccumulation, researchers have recombinantly expressed import-storage systems, which is summarized in Figure 1 and discussed further in the following sections.

### Engineering heavy metal ion import systems

Efforts to improve biological HM uptake have focused on improving uptake from the periplasm into the cytoplasm of Gram-negative bacteria using recombinantly expressed inner membrane importers from three major transporter classes (Transporter Classification Database, TCDB): channels, secondary carriers, and primary active transporters (Saier, 2016). These are summarized in Table 2.

**Table 2:** Transporters used as the import system in bioaccumulation studies.

**Major transporter class**	**Superfamily**	**Family**	**Transporter name**	**Organism**	**Target**
Channels 1.A	Major Intrinsic Protein 1.A.8	/	GlpF/homologs	*Escherichia coli, Corynebacterium diptheriae, Streptomyces coelicolor*	As^3+^
			Fps1	*Saccharomyces cerevisiae*	As^3+^
	Mer 1.A.72	/	MerT/P	*Serratia marcescens, Pseudomonas K-62, Pseudomonas K-12*	Hg
Secondary Carriers 2.A	Transporter-Opsin-G protein-coupled receptors^*^	NiCoT 2.A.52	NixA/homologs	*Helicobacter pylori, Novosphingobium aromaticivorans, Rhodopseudomonas palustris*	Ni/Co
				*Staphylococcus aureus*	Ni/Co
	Major Facilitator 2.A.1	Sugar Porter 2.A.1.1	Hxt7	*Saccharomyces cerevisiae*	As^4+^
		${\text{PO}}_{4}^{2-}$:H^+^ Symporter 2.A.1.9	Pho84	*Saccharomyces cerevisiae*	As^4+^
Primary active transporters 3.A	P-type ATPase 3.A.3	/	MntA	*Lactobacillus plantarum*	Cd
			cdtB/ lp_3327	*Lactobacillus plantarum*	Cd
			TcHMA3	*Thlaspi caerulescens*	Cd
			CopA	*Enterobacter hirae*	Cu

*Transport classification database (TCDB) identifiers are indicated by names where appropriate. Some transporters belong to branches within their superfamily, indicated under the Family column*.

* *A superfamily comprised of transporters from different families*.

#### Channels

Channels (TCDB 1.A) are single component α-helical proteins that can facilitate passive diffusion of HMs according to their concentration gradient across the inner membrane. They are mostly energy-independent, meaning that they do not require the proton-motive force (PMF) or nucleoside triphosphates (NTPs) like ATP and GTP to translocate their substrates (Saier, 2016). For bioaccumulation, researchers have used channels to improve As^3+^ and Hg uptake. For As^3+^, the homotetramer glycerol facilitators (GlpF) from *Escherichia coli* (Singh et al., 2008b, 2010), *Corynebacterium diptheriae* (Villadangos et al., 2014), *Streptomyces coelicolor* (Villadangos et al., 2014), as well as the homolog Fps1 from *Saccharomyces cerevisiae* (Shah et al., 2010) have been used for uptake. These importers belong to the Major Intrinsic Protein superfamily (TCDB 1.A.8). For Hg, the MerT/P transporter from *Serratia marcescens* (Chen and Wilson, 1997; Wilson, 1997; Chen et al., 1998; Bae et al., 2001; Deng and Wilson, 2001; Zhao et al., 2004; Deng et al., 2006, 2008; Deng and Jia, 2011), *Pseudomonas K-62* (Pan-Hou et al., 2002), and *Pseudomonas K-12* (Kiyono et al., 2003) have been tested. Additionally, MerC, MerE, and MerF are other importers that can uptake Hg and although they differ in topology, it is suggested they share the same uptake mechanism (Wilson et al., 2000; Sone et al., 2013). These Mer proteins belong to the Mer superfamily (TCDB 1.A.72).

The zero-energy requirement of small ion channels for uptake makes them appear to be the best choice for bioaccumulating HMs since there is less energetic burden on the cell. However, the rate of this passive uptake is a function of the concentration gradient of the target HM. Once this gradient reaches equilibrium, the GEM can no longer perform bioaccumulation. Although this is not detrimental to removing HMs overall, it must be considered when designing the bacteria to treat wastewater effluents containing relatively high concentrations of HMs. If there are regulatory limits that require the GEM to bioaccumulate against the equilibrium concentration (*viz*. more HM in the cell than in the external environment), an energy-dependent import system *and* storage system are needed. Nearly all studies using channels have used storage systems.

A similar major transporter class are the porins (TCDB 1.B) which use β-barrels to form translocation pathways across the outer membrane of gram-negative bacteria (Reddy and Saier, 2016). In one gene knock-out approach, Schauer et al. determined that FrpB4 channel (UniProt O26042) from *Helicobacter pylori* was involved in Ni uptake. Hence, it may be possible to increase periplasmic HM concentrations by overexpressing divalent cation-selective porins to improve the overall uptake rate from the periplasm into the cytoplasm for storage. It may also be possible to engineer porins with altered HM selectivity using the PoreDesigner workflow (Chowdhury et al., 2018).

#### Secondary carriers

Secondary carriers (TCDB 2.A) are single component proteins that can be further classified as uniporters, symporters, and antiporters (Forrest and Rudnick, 2009; Saier, 2016). For bioaccumulation, symporters have been used to import Ni, Co, and As^4+^. For Ni and Co, NixA from *H. pylori* (Krishnaswamy and Wilson, 2000; Deng et al., 2003, 2005, 2013) and its homologs from *Staphylococcus aureus* (Zhang et al., 2007; Deng et al., 2013)*, Novosphingobium aromaticivorans* (Raghu et al., 2008; Duprey et al., 2014; Gogada et al., 2015), and *Rhodopseudomonas palustris* (Raghu et al., 2008; Gogada et al., 2015) have been used for uptake. These symporters belong to the NiCoT family (TCDB 2.A.52) under the transporter-opsin-G-protein Receptor superfamily with 7 TMS topologies (Yee et al., 2013). For As^4+^, Hxt7 and Pho84 from *Saccharomyces cerevisiae* are used (Shah et al., 2010; Shen et al., 2012). The former is a uniporter belonging to the Sugar Porter Family (TCDB 2.A.1.1) and the latter is a symporter belonging to the PO^4−^:H^+^ Family (TCDB 2.A.1.9), both of which belong to the Major Facilitator Superfamily with 12 TMS topologies (TCDB 2.A.1).

Uniporters depend on the presence of the PMF by using the charge difference across the inner membrane to drive the translocation of positively charged substrates like cationic HMs. Symporters depend on the PMF because they use the protons that generate the charge difference as a co-substrate during uptake of their target substrate. Both secondary carriers thus deplete a portion of the PMF which may impose an energetic burden that negatively impacts growth due to the reduction of protons used by the ATP synthetase to generate ATP. Considering this factor during testing of import system expression levels may minimize growth inhibition, which is often an obstacle during scale-up studies that use propagating cells.

#### Primary active transporters

Primary active transporters (TCDB 3.A) consist of multicomponent protein complexes containing a transmembrane component for the translocation pathway (4–10 TMS), a cytoplasmic energy-coupling ATPase component (~30 kDa) that uses phosphoanhydride bond hydrolysis to drive the translocation of substrates, and sometimes a periplasmic solute-binding component (30–70 kDa) depending on the superfamily. Like secondary carriers requiring the PMF, these importers are able to carry their substrate against a concentration gradient using the hydrolysis of NTPs like ATP and GTP. For bioaccumulation, primary active transporters have been shown to import Cd and Cu. For Cd, MntA, and cdtB from *Lactobacillus plantarum* (Hao et al., 1999; Zagorski and Wilson, 2004; Kim et al., 2005; Deng et al., 2007; Kang et al., 2007) and TcHMA3 from the flowering plant *Thlaspi caerulescens* have been used in uptake (Chang and Shu, 2013). Bioaccumulation studies that used cdtB did not specify its accession number for a database query or any record of the DNA sequence. Based on a differential proteomics study of *L. plantarum* in the absence and presence of Cd, it is suspected that this unknown “cdtB” importer is lp_3327 (UniProt F9UTK4; Ueno et al., 2011). These importers belong to the P-type ATPase superfamily (TCDB 3.A.3). For Cu, CopA from *Enterobacter hirae* has been used in uptake, which is also part of the P-type ATPase superfamily (Zagorski and Wilson, 2004).

By using cellular ATP reserves, these primary active transporters directly consume chemical energy, which likely imposes a heavier energetic burden on the bacteria. Similar to secondary carriers, researchers ought to be aware of this relationship between bioaccumulative capacity and cellular growth due to the burden of HM import if they are considering scale-up efforts. Interestingly, another large class of primary active transporters has seen no attention in bioaccumulation studies: ABC transporters (TCDB 3.A.1). There are three classifications of ABC importers: Type I, Type II, and ECF-type. Many microorganisms, especially pathogenic species, have evolved Type I and II ABC importers with high affinities for HMs. For example, *Yersinia pestis*, the cause of the bubonic plague, possesses the *YntABCDE* operon which encodes a highly specific Ni ABC importer (Sebbane et al., 2002). Some pathogens encode a gene for solute-binding components that use biosynthesized metallophores capable of scavenging metals by binding them with picomolar affinities. This prevents the metal from being captured by the host cells' transporters which is a defense mechanism to minimize the pathogens' virulence. Yersinopine from *Y.pestis*, staphylopine from *S. aureus*, and pseudopaline from *Pseudomonas aeruginosa* have been identified as metallophores that enable uptake of metals from metal-poor environments like the human respiratory tract (McFarlane et al., 2018). This high degree of specificity may allow for the bioaccumulation of valuable metals present at very low concentrations in wastewater. The majority of research in ABC transporters is directed at understanding the role of ABC transporters in infections and diseases (Remy et al., 2013; Singh et al., 2013; Fischer et al., 2016), as well as how they facilitate multidrug resistance (Mousa and Bruner, 2016). This provides a strong molecular understanding of ABC transporters that positions them for testing in bioaccumulation studies.

### Engineering heavy metal ion storage systems

Efforts to improve the HM storage in bacteria have focused on the production of cytoplasmic metal-binding entities for sequestration of HMs to minimize poisoning from oxidative stress. These entities are mostly metal-binding proteins (MBPs), but also include enzymes that produce peptides and other polymers that can also bind to HMs. Studies that explicitly explored the use of these MBPs and enzymes for bio-HMR are summarized in Tables 3, 4. Table 3 is not an exhaustive list of all proteins used to sequester HMs due to the vast body of literature on metallothioneins (MTs).

**Table 3:** Metal-binding proteins and soluble fusion partners used as the storage system in bioaccumulation studies.

**Type**	**Species**	**Name**	**UniProt ID**	**Fusion partner**	**Target**	**References**
Bacteria	*Corynebacterium glutamicum* ATCC 13032	MT	A0A068BCQ0	–	Multimetal	Jafarian and Ghaffari, 2017
	*Escherichia coli*	ArsD	P46003	–	As^3+^	Villadangos et al., 2014
		ArsR	P37309	–	As^3+^, MMA, DMA	Yang et al., 2013; Villadangos et al., 2014
			P37309	ELP153, elastin-like protein made of 153 repeats of VPGVG	As^3+^	Kostal et al., 2004; Shah et al., 2010
Fungi	*Neurospora crassa*	cmt	P02807	lpp, major OM prolipoprotein (P69776)	Multimetal	Romeyer et al., 1990
				araB', truncated ribulokinase (P08204)	Multimetal	Romeyer et al., 1990
				MBP, maltose binding protein	Cd	Pazirandeh et al., 1995
	*Saccharomyces cerevisiae*	MT (unspecific)	–	GST, glutathione-S-transferase	Hg	Wilson, 1997
		MT (unspecific)	–	GSS, glutathione synthetase	Cd	Kim et al., 2005
Plant	*Arabidopsis thaliana*	MT1A, MT1C, MT2A, MT2B, MT3, MT4A, MT4B	P43392, Q38804, P25860, Q38805, O22433, P93746, Q42377	myrGFP, myristoylated green fluroescent protein	Multimetal	Ruta et al., 2017
	*Fucus vesiculosus*	MT	O96717	MBP, maltose binding protein	As^3+^	Singh et al., 2008b
	*Halostachys caspica*	MT	W6AWJ0	Trx, thioredoxin	Multimetal	Liu et al., 2016
	*Noccaea caerulescens*	MT1, MT2a, MT2b, MT3	A9UKL0, C5HGF0, C5HGE1, C5HGE7	myrGFP, myristoylated green fluroescent protein	Multimetal	Ruta et al., 2017
	*Oryza sativa (japonica)*	MT-I1b, MT-I2b, MT-I3a, MTII-1a	Q10N03, Q5JM82, A1YTM8, XP_015614224 (NCBI)	GST, glutathione-S-transferase	Hg	Shahpiri and Mohammadzadeh, 2018
	*Pisum sativum*	MTA	P20830	GST, glutathione-S-transferase	Ni, Co, Hg	Chen and Wilson, 1997; Wilson, 1997; Chen et al., 1998; Krishnaswamy and Wilson, 2000; Deng and Wilson, 2001; Deng et al., 2003, 2005, 2006, 2008; Zhao et al., 2004; Deng and Jia, 2011; Tiwari et al., 2011
Animal	*Ascidia sydneiensis*	Vanabin1, 2	Q86BW3, Q86BW2	MBP, maltose binding protein	Cu	Ueki et al., 2003
	*Homo sapiens*	MT1A	P04731	MT1A (oligomeric)	Cd, As^3+^	Ma et al., 2011
				GST, glutathione-S-transferase	Cd, As^3+^	Su et al., 2009; Ma et al., 2011
				MBP, maltose binding protein	Multimetal	Kao et al., 2006
		MT2A	P02795	araB', truncated ribulokinase (P08204)	Cd, Cu	Romeyer et al., 1988
				β-galactosidase	Cd	Wilson, 1997; Yoshida et al., 2002
				GFP, green fluorescent protein	Cu	Geva et al., 2016
		MT4	P47944	gusA, β-glucoronidase (P47944)	Cd, Cu	Sriprang et al., 2002
	*Longpotamon (Sinpotamon) honanense*	Mt	F8UU34	SUMO, small ubiquitin modifier	Multimetal	He et al., 2014
	*Mus musculus*	Mt1	P02802	GST, glutathione-S-transferase	Hg	Ruiz et al., 2011
				MBP, maltose binding protein	Multimetal	Kao et al., 2006
	*Oreochromis mossambicus*	mt	P52726	MBP, maltose binding protein	Multimetal	Kao et al., 2006
	*Ovis aries*	MT2	P68302	MBP, maltose binding protein	Multimetal	Sauge-Merle et al., 2012
	*Pheretima aspergillum*	MT2	C1IE33	Trx, thioredoxin	Cd	Gong et al., 2015
Synthetic		EC20	–	–	Hg	Bae et al., 2001

*Multimetal indicates when researchers have tested for bioaccumulation of more than two heavy metals in a single publication*.

**Table 4:** Phytochelatin synthesis enzymes used in storage systems for bioaccumulation studies.

**Type**	**Species**	**Name**	**UniProt ID**	**Target**	**Source**
Bacteria	*Escherichia coli*	GshI/GshA^*^	A7ZQC1^*^ or EG10418 (NCBI)	Cd	Kang et al., 2007
Fungi	*Schizosaccharomyces pombe*	SPAC3H1.10 (PC synthase)	Q10075	Cd, As^3+^	Kang et al., 2007; Singh et al., 2010
Plant	*Arabidopsis thaliana*	PCS1	Q9S7Z3	Cd, Multimetal, As^3+^	Sauge-merle et al., 2003; Sriprang et al., 2003; Singh et al., 2008a; Shah et al., 2010
	*Ceratophyllum demersum*	PCS1	E5GCW5	Multimetal	Shukla et al., 2013
	*Nicotania tobacum*	PCS1	AY235426 (NCBI)	Multimetal	Young et al., 2005
	*Pyrus calleryana*	PC synthase	S5UK20	Multimetal	Li et al., 2015
	*Thlaspi caerulescens*	cysE	EG10187 (NCBI)	Cd	Chang and Shu, 2013
		GshA	EG10418 (NCBI)	Cd	Chang and Shu, 2013
		GshB	EG10419 (NCBI)	Cd	Chang and Shu, 2013
		PC synthase	AY540104.1 (NCBI)	Cd	Chang and Shu, 2013

GshA^*^ is the mutant version of GshA that is insensitive to negative feedback inhibition. Multimetal indicates when researchers have tested for bioaccumulation of more than two heavy metals in a single publication.

#### Genetically encoded metal-binding proteins

Ligands that provide binding sites for storage in bioaccumulation studies have mostly come from genetically encoded MBPs. The largest group of proteins used as storage systems are MTs, a polyphyletic superfamily of MBPs that have been studied since 1957 when the horse kidney MT was discovered (UniProt P02801; Margoshes and Valiee, 1957). MTs are ubiquitous because they are found in prokaryotes, archaea, and eukaryotes. However, their evolutionary relationships are obscure, so it has been suggested that they are products of convergent evolution (Capdevila and Atrian, 2011). Since MTs do not have a single common ancestor, the features used to help identify them include low molecular mass, characteristic amino acid composition (i.e., high CxC and CC motifs), and spectroscopic characteristics indicative of metal-thiolate bonds (Maret and Wedd, 2014). These cysteine residues are necessary for sequestration because they can strongly coordinate the HMs. The majority of MT research focuses on their abilities to bind to zinc, Cd, and Cu, but it is apparent that MTs can also bind to Hg, As^3+^, Ni, and Co based on bioaccumulation studies (Table 1).

Researchers realized early on that aggregation of overexpressed MTs can reduce their effective storage capacity (Irons and Crispin Smith, 1976). MTs have since been fused to a variety of soluble fusion partners, including the popular maltose-binding protein and glutathione-S-transferase (Table 3). Although successful, many bioaccumulation studies that use MTs as the storage system fail to report a control where only the fusion partner is expressed. This is important as it reveals the extent to which these fusion partners participate in HM sequestration. It might be unclear whether the MT makes a difference given the relative size comparisons between a 5 kDa MT and a 43 kDa maltose-binding protein that may adsorb HMs to its surface. Expressing the fusion partner alone, especially larger ones like maltose-binding protein, may deplete material resources (i.e., amino acids) needed for other cellular processes, therefore negatively impacting growth.

Additionally, bacteria maintain a redox environment in the cytoplasm that inhibits disulfide bridge formation in cytoplasmic proteins (Raina and Missiakas, 1997; Bessette et al., 1999). By overexpressing these cysteine-rich MTs, an increase in demand for cysteine and methionine for biosynthesis of other endogenous proteins may alter this homeostasis and negatively impact growth. An alternative to the cysteine-rich MTs are histidine-rich MBPs. These proteins have recently been discovered and found to function as natural HM storage systems that play crucial physiological roles in metal homeostasis. For example, Hpn (UniProt P0A0V6) from *H. pylori* has been characterized as an HM storage protein that can reversibly bind Ni (Gilbert et al., 1995; Ge et al., 2006; Saylor and Maier, 2018). SCO4226 (UniProt Q9FCE4) from *Streptomyces coelicolor* A3 has also been characterized as a Ni storage protein (Lu et al., 2014). These discoveries suggest there are numerous undiscovered HM storage systems that have been evolved to retain HMs in intracellular spaces, perhaps better than MTs which are normally used as a stress response. This is a potential avenue in bioaccumulation research.

#### Enzymatically produced metal-binding peptides and polymers

HM storage can also be mediated through small polymers that are enzymatically produced from materials readily available in the cytoplasm (summarized in Table 4). The most common small polymer is phytochelatin, a chain of glutathione (GSH) produced from ligating L-cysteine and L-glutamate to form γ-glutamylcysteine (γEC), followed by another ligation between L-glycine and the γEC. The first step requires ligase GshI (EC 6.3.2.2) and the second step requires another ligase GshII (EC 6.3.2.3), which are both ATP-dependent enzymes. Up to eleven γECs can be sequentially added to the growing GSH chain using phytochelatin synthase (PCS; EC 2.3.2.15), which is commonly found in plants that possess HM resistance (Grill et al., 1985; Singh et al., 2010). PCS alone can be sufficient for bioaccumulation, but can be made more effective through metabolic engineering to increase the pool of phytochelatin precursor compounds cysteine, γEC, and GSH by overexpressing cysE, GshI, and GshII, respectively (Table 4). Earlier studies identified GSH production as the bottleneck in the pathway for phytochelatin production, so a mutant GshI insensitive to feedback inhibition was discovered and later used in bioaccumulation studies (Murata et al., 1983).

Another storage system uses the production of polyphosphate (polyP) using polyphosphate kinase (EC 2.7.4.1) from *Klebsiella pneumonia* (UniProt Q07411). PolyP has only been used to bioaccumulate Hg, but has also been reported as a natural response to As^3+^, Cu, and Ni exposure which suggests polyP may be used for storing other HMs (Gonzalez and Jensen, 1998; Alvarez and Jerez, 2004; Seufferheld et al., 2008). In contrast, an alkaline phosphatase (phoK, EC 3.1.3.1) from *Sphingomonas* sp. (UniProt A1YYW7) can hydrolyze phosphoric monoesters from biomolecules into single phosphate ions that precipitate U (VI) as uranyl phosphate (Kulkarni et al., 2013).

Beyond phytochelatins and polyP, some organisms naturally respond to HM exposure by upregulating enzymes that produce amino acids and organic acids to chelate/complex HMs. For example, *Acidithiobacillus ferrooxidans* ATCC 23270 was shown to upregulate expression of its histidine biosynthesis operon when exposed to 40 mM CuSO_4_ (Almárcegui et al., 2014). The authors suggested that this upregulation may increase the pool of cytoplasmic histidine to chelate Cu ions to prevent oxidative damage. A similar upregulation and speculation were noted in the same study for enzymes that participate in the cysteine biosynthesis pathway. In plants, the overproduction of organic acids like citrate, maleate, and oxalate have been reported during HM exposure (Clemens, 2001; Hall, 2002). This is another potential avenue in bioaccumulation research.

### Overarching inconsistencies

There are three systemic problems in bioaccumulation studies. First, the emphasis on reaching nearly 100% removal of HMs from effluent samples may have led some researchers to overlook the importance of washing the HM-saturated cells with chelators like EDTA or dilute acid to distinguish between the contributions of biosorption vs. bioaccumulation. Washing with phosphate buffer saline (PBS), Luria-Bertani (LB) growth media, and water are indeed general-purpose wash solutions, but they may not sufficiently remove HMs adhered to the surface of the cell, leading to overestimations of the bioaccumulative performance. Second, there is a lack of consensus in units for reporting bioaccumulative performance. Researchers report the amount of HMs (numerator) as μmol_x_ or mg_x_, and although difficult to compare molarity and mass units at first glance, they can be interchangeable given the molecular weight. However, some researchers report the amount of biomass (denominator) as number of cells or optical density units instead of mass units, which precludes reliable comparison of bioaccumulative performance since they cannot be accurately converted to mass units. Lastly, GenBank accession numbers, UniProt IDs, or the actual annotated sequences in supplementary materials to access the DNA or amino acid sequences is often obscurely described and sometimes absent from papers, which makes it more difficult to perform bioinformatic analyses and to replicate experiments. Establishing wash protocols (EDTA/ dilute acid wash), agreed-upon bioaccumulative capacity units (mg_x_ per g_dry weight_), and taking steps to increase transparency in the genetic constructs used are necessary for advancing bioaccumulation research.

## Re-thinking bioaccumulation

Bioaccumulation research began in the early 1990s when environmental stewardship grew in importance, thus resulting in the paradigm of this bio-HMR technology to be centered around bioremediative applications. Most studies focused on genetically engineering bacteria to import and store HMs such that the concentration of HMs in the simulated and real wastewater effluent would decrease below regulatory limits. The goal was to maximize bioaccumulative capacity to treat metal pollution rather than optimizing the GEM's overall performance so that practical implications could be considered. Although achieving 100% removal has been foundational to understanding what strategies work for capturing specific HMs, it does not address two grand challenges, as pinpointed by proponents of biosorption: (1) bioaccumulation is slow and (2) bioaccumulation is irreversible (Vijayaraghavan and Yun, 2008; Kuroda and Ueda, 2011).

Bioaccumulation is not like biosorption where dead bacteria can still remove HMs. Heterologous import-storage systems require GEMs to be alive so they are able to recombinantly express the necessary proteins while surviving in wastewater effluents not conducive for sustaining and propagating itself. It is especially important that the GEMs are alive because the uptake machinery requires renewal of the PMF and NTPs to power HM translocation into the intracellular space for sequestration. Additionally, bioaccumulation is not like biosorption where an acid wash can release HMs from the exterior surface of microorganisms, allowing them to be used in another round of biosorption. In bioaccumulation, the cell wall and lipid membrane need to be physically or chemically disrupted to acquire the HMs, meaning the cells cannot be reused.

Advancing bioaccumulation research may benefit from a paradigm change. Rather than using bioaccumulation as a bioremediative tool, it could be used as a “bioextractive” tool for removal *and recovery* of HMs from wastewater effluents. Nearly all bioaccumulation studies reviewed here have focused on the former, as discussed in the following sub-sections, but these efforts pave way for the latter. Reframing bioaccumulation as a bioextractive tool that could participate in the metal supply chain de-emphasizes the importance of reducing HM concentrations below regulatory limits, and would instead focus on addressing obstacles with practical implications: scaling bioaccumulation to an industrial-scale process, transferring import-storage systems to non-model organisms, and expanding the currently limited selection of importer-storage systems. This review argues that overcoming these obstacles can bring bioaccumulation closer to being adopted by industries.

### Scale-up

**Figure 2:**
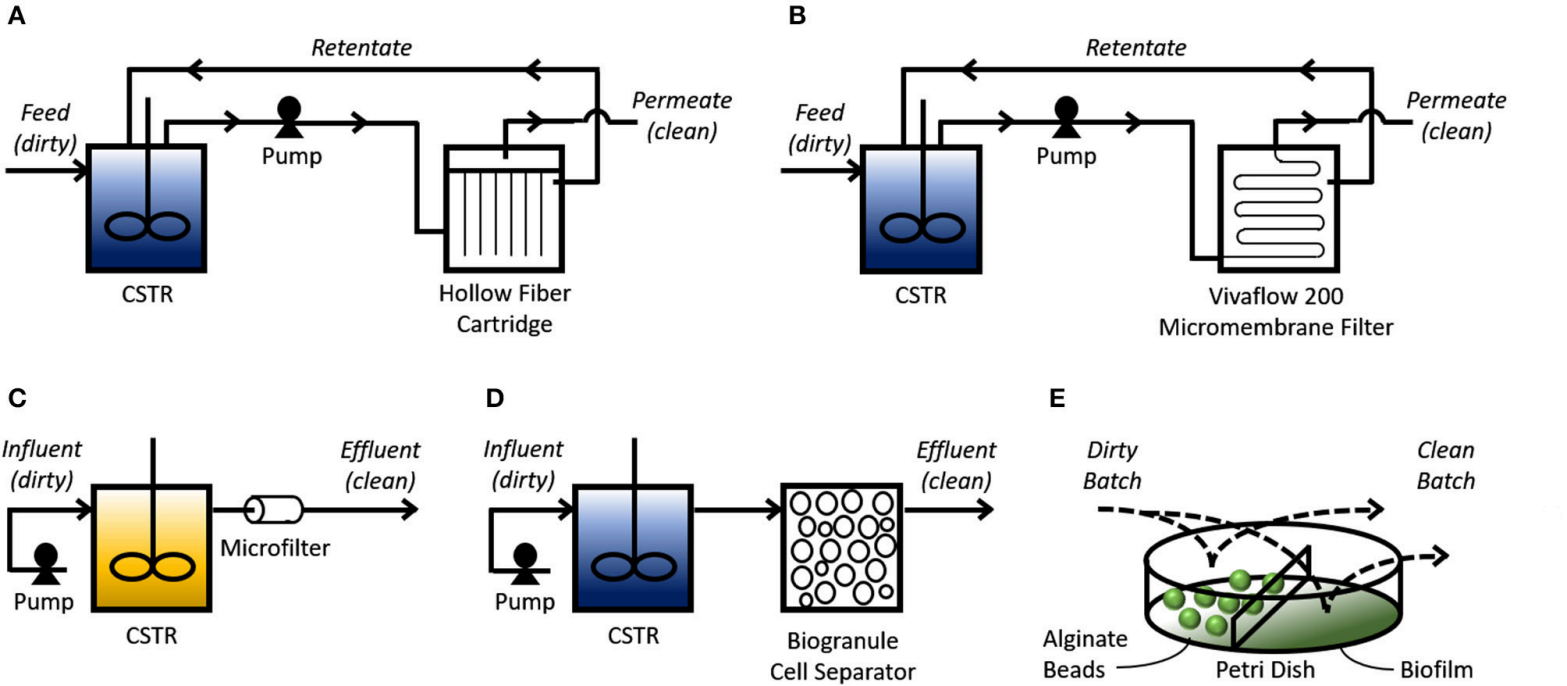
Bioprocess schemes for bioaccumulation. Continuous bioprocesses **(A–D)** use a continuously-stirred tank reactor (CSTR) to mix influent (“dirty” simulated or real wastewater effluent) with the bioaccumulative GEMs. **(A)** A pump transfers the mixture into an HF cartridge where the pressure pushes solution through the tubular filtration membrane, depicted as vertical lines, which can then leave as clean permeate. HM-saturated GEMs are physically separated from the clean permeate as they are too big to pass through this membrane. Solution that did not pass through the membrane can leave the HF cartridge as retentate and return to the reservoir. **(B)** Similar to A, except a Vivaflow 200 micromembrane filter is used to separate the clean permeate from the HM-saturated cells in the retentate, which returns to the CSTR. **(C)** A pump transfers influent mixed with growth media into the CSTR to be mixed with cells that are trapped in the tank using a 0.45 μm pore-diameter nitrocellulose filter. This CSTR provides conditions for cell propagation. Solution coming out from the filter is cleaned effluent mixed with spent growth media. **(D)** Similar to C, except cells leaving the CSTR are sorbed onto silica granules to separate them from the clean effluent. Batch bioprocesses **(E)** allow an influent sample to mix with cells entrapped in alginate beads, or a biofilm, to remove HMs. Process flow diagrams are adapted and simplified from their corresponding studies. Blue CSTRs indicate cells resuspended in non-growth media; yellow CSTR indicated cells resuspended in growth media.

Lab-scale experiments provide preliminary results that demonstrate the underlying molecular mechanism (i.e., engineered import-storage systems) can consistently perform bioaccumulation. However, suggesting from these small-scale tests that bioaccumulation is more cost-effective and environmentally friendly than existing HMR technologies is short-sighted and speculative at most if scale-up tests and technoeconomic-environmental risk assessments have not been successfully completed. Scale-up studies reveal issues that arise from increasing dimensions: transport phenomena, shear impact, and genetic stability, among other issues (Reisman, 1993). The majority of bioaccumulation studies use shake flask experiments that incubate GEMs with HMs. These miniature batch experiments are the first step to scaling up bioaccumulation to treat industrially relevant volumes of wastewater. Researchers have taken further scale-up steps by designing bioprocesses that utilize filtration membranes, biobeads, and biofilms (Figure 2).

#### Continuous bioprocesses

The Wilson Group at Cornell University spearheaded the development of continuous bioprocesses for treating simulated and real wastewater effluents using bioaccumulative (genetically engineered) *E. coli*. Their earlier designs used membrane bioreactors (MBRs) in a recycle-purge process. A hollow-fiber (HF) cartridge provided a 3-D matrix for immobilization of cells after they were pumped out of the mixing tank. Their first study was able to show through mathematical models and experimental validation that their HF-MBR could reduce a 2 g/L Hg influent down to 6.3 ng/L Hg (Chen et al., 1998). A follow-up study using real wastewater (origins unspecified) showed the HF-MBR could treat 16.3 L of wastewater effluent containing 2.58 mg/L Hg before they started to observe significant amounts of Hg in the effluent released from the HF cartridge (Deng and Wilson, 2001). As the import-storage system, both studies used the *Serratia marcescens* MerT/P transporter and the *Pivum sativum* MT expressed in *E. coli* (Figure 2A). A later study replaced this channel with the P-type ATPase MntA from *Lactobacillus plantarum* to remove Cd (Kim et al., 2005). Instead of an HF cartridge for immobilization, this study used a Vivaflow 200 micromembrane filter where the simulated Cd-laden wastewater effluent and bacteria could mix together in a reservoir for bioaccumulation to occur. This mixture was then slowly pumped through the filter for cleaned effluent (permeate) to be collected; the Cd-saturated bacteria (retentate) would be concentrated over time in the tank. This design was able to reduce 5 L of 1 mg/L Cd influent to 0.2 mg/L Cd (Figure 2B). In these three experiments, the cells were first cultivated until they reached a certain OD_600_ for IPTG induction. After an induction period, the cells were harvested, pelleted, resuspended in phosphate buffer, then transferred to the continuously-stirred tank reactor (CSTR) for continuous bioaccumulation.

Deng et al. (2008) later tested whether it was possible to maintain cells in LB growth media, thus eliminating the need for resuspension in phosphate buffer. Using a CSTR, they cultivated their cells to stationary phase under IPTG induction conditions, then pumped Hg-laden wastewater effluent mixed with LB into the CSTR. These cells used the same MerT/P transporter and MT from their early work with the HF-MBR (Deng et al., 2008). Rather than immobilizing cells to an HF cartridge, effluent leaving the CSTR would pass through a 0.45 μm-pore-diameter nitrocellulose microfilter to prevent the cells from escaping. With a dilution rate of 0.36 h^−1^, this CSTR was able to reduce a 2, 4, and 8 mg/L Cd influent to 0.2, 0.51, and 1.13 mg/L Cd, respectively (Figure 2C). One study from a different group proposed a process flow diagram where a CSTR is also used to mix the GEM with cobalt-laden wastewater, but instead of using a microfilter to keep the saturated bacteria in the tank, they suggested gradually pumping out the Co-saturated GEMs for sorption onto silica granules (Figure 2D; Raghu et al., 2008).

#### Batch bioprocesses

Bacterial immobilization allows the user to physically separate the HM-saturated cells from the solution they are treating. HF cartridges used in the continuous bioprocesses as previously mentioned are examples of immobilization. Alternatively, other researchers used a batch bioprocess where cells entrapped in alginate beads were soaked into simulated wastewater effluent for bioaccumulation to occur. Kiyono et al. (2003) created a strain of *E. coli* expressing the *merR-o/p-T-P-B1* gene for the mercury uptake channel, an organomercurial lyase to convert methylmercury to the less toxic inorganic Hg ion, and polyphosphate kinase for polyP-based storage. Using 3 g of alginate-entrapped bacteria, they were able to reduce 5 mL of a 10 μM Hg solution to 0.2 μM (Figure 2E). Similarly, Kulkarni et al. genetically engineered *Deinococcus radiourans* to overexpress a *phoK* gene encoding an alkaline phosphatase (EC 3.1.3.1). By entrapping these cells in alginate beads and soaking them in a 1 mM uranyl carbonate/5 mM β-glycerophosphate solution, they were able to precipitate ~90% of U in solution as chernikovite (Figure 2E; Kulkarni et al., 2013).

Another approach for immobilizing biomass is biofilm formation on a solid support. One study engineered *E. coli* to express a NiCoT permease for Ni uptake, and a synthetic curli operon that allowed cells to adhere to polystyrene (Duprey et al., 2014). By growing this strain (S63) in polystyrene Petri dishes, they created a biofilm that could be soaked in a Ni-laden solution. This approach was able to remove 4.8 and 6 mg/g_dry weight_ from 50 μM Ni to 50 μM Co solutions, respectively (Figure 2E). As suggested in this study, biofilm formation is particularly useful because it can also increase the cells' resistance to pollutants and, as noted elsewhere, can be engineered for higher robustness and more diverse metabolic activities to allow for better growth (Hays et al., 2015).

### Chassis selection

Most bioaccumulation studies used *E. coli*, and occasionally *S. cerevisiae*, to test the import-storage systems' bioaccumulative capacity. These model organisms have been thoroughly studied from multiple perspectives like molecular biology, cellular physiology, and bioinformatics; this makes them the best “chassis” for prototyping import-storage systems (Blount, 2015; Liti, 2015). However, *E. coli* and *S. cerevisiae* have optimal growth conditions that allow them to thrive. Large deviations from these conditions can negatively impact their bioaccumulative performance since they must adapt to environmental changes, which requires energy that would otherwise be used to express and power the import-storage systems (Scott et al., 2010; Wu et al., 2016). Most wastewater effluents are not conducive for sustaining and propagating these model organisms because their pH, salinity, temperature, dissolved oxygen, redox potential, radioactivity, and overall cleanliness (i.e., organic pollutants and suspended solids causing shear damage) are non-optimal. To treat real wastewater effluents, it is necessary to transfer these import-storage systems to non-model organisms that are aligned with several practical considerations (Adams, 2016).

A major consideration is the chassis' compliance with environmental regulations. *E. coli* is not generally recognized as safe (GRAS) by the US FDA. In accordance, a GlpF channel and ArsR/D was used to bioaccumulate As^3+^ and As^4+^ using *Corynebacterium glutamicum*, which is classified as GRAS (Meiswinkel et al., 2013; Villadangos et al., 2014). These considerations are important due to the negative public perception of GMOs used in biotechnologies that often deters companies from adopting them (Małyska et al., 2017).

The nature of the wastewater effluent is important. Effluents from metal refinery industries and mines often contain low levels of organic matter that could serve as a carbon source for heterotrophic growth by *E. coli*. One study genetically engineered the photosynthetic bacterium *Rhodopseudomonas palustris* to express the MerT/P channel and *P. sativum* MT for Hg bioaccumulation (Deng and Jia, 2011). *R. palustris* is capable of alternating between four modes of metabolism, two of which are relevant: photosynthetic and chemoautotrophic (Nelson and Fraser, 2004). This versatility potentially minimizes the need to dose additional nutrients in the influent since *R. palustris* can survive with light, air, and trace micronutrients already present in the wastewater effluent. Elsewhere, effluents from nuclear power reactors can be radioactive. The extremophilic (radiation-resistant) bacterium *Deinococcus radiodurans* has been genetically engineered to bioaccumulate Co (Gogada et al., 2015) and bioprecipitate U (uranyl carbonate) from simulated radioactive wastewater effluent (Kulkarni et al., 2013). This species' naturally extreme resistance to radioactivity, up to 6.4 kGy, conferred a major advantage over *E. coli* which could only tolerate 20 Gy. Genetically engineering extremophiles to bioaccumulate HMs from the very harsh conditions imposed by some wastewater effluents is reviewed elsewhere and very important for industrial adoption (Marques, 2018).

The location of the wastewater effluent is also important. *Mesorhizobium huakuii subsp*. Rengei B3 has been engineered to express a homotetramer human MT (MTL4) and a PCS-based storage system (Sriprang et al., 2002, 2003). This symbiont grows slowly in soil but can infect the flowering plant *Astragalus sinicus* to form N_2_-fixing root nodules. Their rationale for using *M. huakuii* as the chassis was so that it could clean HM-contaminated rice fields *in situ* during the idle periods where the *A. sinicus* plant would normally be grown to fertilize the soil. Removal of the plant along with the HM-saturated root nodules would therefore clean the soil. *M. huakuii* has a doubling time of 4–6 h, whereas *E. coli*'s is 20 min (Sezonov et al., 2007; Nandasena et al., 2009). This is an example where *slow* bio-HMR is appropriate and desirable. Using *M. huakuii* as the chassis allows bioaccumulation to occur on time scales closer to the rate at which root nodules are formed. In this scenario, rapid biosorption of HMs to *M. huakuii* would likely deteriorate its ability to propagate into more cells that could participate in bio-HMR.

Import-storage systems are essentially genetic circuits that have thus far used chemical signals (input) to induce expression of the import-storage machinery for bioaccumulation (output; Zhang and Jiang, 2010; Brophy and Voigt, 2014). The genetic regulatory elements that initiate transcription and translation of these systems in the *model* organisms may not function in the *non-model* organisms, like those described here. This is because other microbial species may use different machinery to express their proteins, and consequently, the user may need to change the promoters, ribosome binding sites, terminators, and other genetic regulatory elements (Kushwaha and Salis, 2015). Developing standardized synthetic biology toolboxes by characterizing genetic regulatory elements from non-model organisms will be paramount for treating a larger variety of wastewater effluents. For example, efforts to develop this toolbox for *Acidithiobacillus ferrooxidans* may open vastly untapped opportunities to remove and recover HMs from acidic wastewater effluents (pH 1–2), especially acid mine drainage which by nature can be polluted with HMs from upstream mining processes (Kernan et al., 2016; Gumulya et al., 2018).

### “Omics” and protein design

Researchers tend to use the same import-storage system for bioaccumulating HMs once it has been shown to work. This is beneficial as it demonstrates experimental reproducibility which helps with evaluating the feasibility of scale-up studies. For example, only NiCoT permeases and *P. sativum* MTs were used in Ni bioaccumulation. Channels from the *Mer* operon are used for all Hg import systems, and P-type ATPases are the only class of transporters used in Cd import systems. However, a lack of diversity in import-storage systems can constraint users to a narrow range of options that prevents them from treating a larger variety of wastewater effluents.

Microorganisms will respond to HM exposure by changing gene expression such that the cell minimizes oxidative damage to cellular components. Metallomics can uncover novel import-storage systems by studying which genes are expressed differently when an organism is exposed to a HM (Haraguchi, 2004). For example, downregulated gene expression may include transporters normally expressed to uptake metals needed for cellular processes and upregulated gene expression may include MBPs intended for intracellular sequestration of HMs. As noted earlier, Almárcegui et al. (2014) used LC ESI-MS/MS with ICPL to compare the proteomes of *Acidithiobacillus ferrooxidans* ATCC 23270 in the absence and presence of Cu and discovered that it upregulates enzymes from the histidine and cysteine biosynthesis pathways. A similar study for this strain used DIGE and MALDI-TOF/TOF MS with Refraction-2D labeling for cultures in the absence and presence of 500 mM U, and uncovered four upregulated uncharacterized proteins. AFE_2018 was proposed to be a putative MBP, and AFE_1839, AFE_2599/AFE_3116, AFE_2600/AFE3117 were suggested to be involved in sulfur metabolism with a potential role in the biosynthesis of cysteine-rich MTs (Dekker et al., 2016). Elsewhere, Zhai et al. (2017) used LC/LC-MS/MS with iTRAQ labeling and discovered a putative Cd transporter lp_3327 from *Lactobacillus plantarum* that was downregulated in the presence of 5 mg/L Cd, which suggests it could be used in an import system. Lastly, Zammit et al. (2016) used DIGE with CyDyes labeling for *Cupriavidus metallidurans* CH34 grown in the absence and presence of gold, and discovered upregulated putative gold-binding proteins like CupC (Zammit et al., 2016). CupC could potentially function as a storage system for gold bioaccumulation, which raises an important question: can GEMs bioaccumulate precious and strategic metals? This has large implications in bioextraction and is a potential avenue in bioaccumulation research.

Transportomics research is nascent, but can already provide insight regarding the energetics of transporters used in import systems. Part of the grand challenge of bioaccumulation being slow is the gratuitous use of cellular energy to power the import system rather than sustaining and propagating the cell. Therefore, the energy requirements of an importer ought to be considered when choosing which to use for the import system. It is hypothesized that there could be an evolutionary pressure to select for low energy transporters, such as channels and secondary carriers (Darbani et al., 2018). By analyzing the transportomes of myriad organisms across the tree of life, they found that the eukaryotic transportomes evolved to favor secondary carriers and channels over the primary active transporters. This suggests it might be energetically more favorable to not use primary active transporters in bioaccumulation, but it may also point to the need to mutate these transporters to use less cellular energy. Directed evolution has been used to mutate NTP-binding domains of ABC transporters to alter their transport efficiency (Eom et al., 2005; Low et al., 2010).

Instead of discovering HM transporters and MBPs through “omics” approaches as described above and elsewhere (Yu et al., 2012; Ziller et al., 2017), a user may also leverage the metal-binding promiscuity of these proteins to design new import-storage systems with different HM specificity and selectivity (Pordea, 2015). Directed evolution is a traditional methodology in protein engineering that has been used to alter the substrate specificity of the PnuC transporter. PnuC's natural substrates are nicotinamide ribosides, but by coupling its uptake activities to a synthetic thiamine pyrophosphate-dependent riboswitch controlling the expression of an antibiotic marker gene, they were able to screen a library for mutants able to uptake thiamine, which is structurally different from nicotinamide ribosides. HM riboswitches exist and could be used to synthetically select for transporters able to import heavy metals currently unexplored in bioaccumulation research (Furukawa et al., 2015; McCown et al., 2017).

A newer methodology in protein engineering uses ancestral sequence reconstruction (ASR) to search for ancient proteins with broader substrate ranges as templates for grafting new proteins with directed evolution (Gumulya and Gillam, 2017). ASR has been used to uncover an ancestral amino acid binding protein (AABP) that could bind both L-arginine and L-glutamine, but eventually gave rise to L-glutamine-specific importers in the ABC transporter superfamily (Clifton and Jackson, 2016). From this, there may be ancient HM transporters with promiscuous transport activity that could serve as templates in directed evolution with HM riboswitches to evolve new HM transporters. Storage systems may also benefit from protein engineering efforts where researchers have intensively focused on engineering metal-binding sites (Cherrier et al., 2012; Valasatava et al., 2015; Akcapinar and Sezerman, 2017). Such efforts may use existing MBPs as templates to produce “cysteine-free” storage proteins that are intrinsically soluble. This could reduce the impact of the storage system on cellular material resources by circumventing the need for soluble fusion partners.

Lastly, artificial “designer proteins” incorporate unnatural amino acids (UAAs) into their structure, thus conferring unnatural functions. This is based on genetic code expansion where an orthogonal aminoacyl-tRNA synthetase and a tRNA carrying this UAA can incorporate it when a UAG codon, normally a stop codon, is reached during mRNA translation (Davis and Chin, 2012; Dien et al., 2018). Mills et al. (2013) used a Rosetta design methodology to create a metalloprotein containing (2,2'-bypyridin-5yl)alanine that was able to bind to divalent cations like Fe^2+^, Ni^2+^, and Co^2+^ based on x-ray crystallography and fluorescence data. The use of UAAs is especially important for the storage of metal species that may have unusual electronic properties that natural amino acids may not easily coordinate. Artificial metalloprotein design methodologies are described elsewhere (Lin, 2017; Schwizer et al., 2018; Yu et al., 2018).

## Outlook

Bioaccumulation has been developed as a bio-HMR technology for bioremediation. Researchers have designed GEMs to uptake HMs using channels, secondary carriers, and primary active transporters. These GEMs have also been designed to sequester HMs using metallothioneins, phytochelatins, and polyPs. This review argues that GEMs can provide a robust and versatile platform for the removal (bioremediation) and recovery (bioextraction) of HMs from wastewater effluents. By organizing how researchers have enhanced the uptake and sequestration of HMs in bioaccumulative GEMS using recombinantly expressed import-storage systems, gaps in knowledge and unexplored potential avenues of research are delineated. To advance bioaccumulation research, developments at the cellular and bioprocess level are recommended.

### Cellular level

There is a poor understanding of how an active import-storage system affects the host cell's metabolism (i.e., availability of energy and material resources). Similarly, how the expression of cysteine-rich MTs fused to large soluble partners affects the host cell's metabolism during bioaccumulation is also poorly studied. A stronger understanding of these dynamic relationships is needed to optimize the expression of import-storage systems using synthetic biology tools so that GEMs can compete with native wildtype microorganisms while bioaccumulating HMs. Unexplored potential avenues for future bioaccumulation studies include using outer membrane pores, ABC transporters, histidine-based storage proteins, and biosynthesized small molecule chelating/ complexing compounds. In addition, using extremophiles like *A. ferrooxidans* and *D. radiourans* to treat harsh wastewater effluents, discovering new import-storage systems with omics approaches, and modifying their specificity and selectivity through protein engineering methodologies are largely unexplored areas despite their potential to create GEMs more suited to real-world needs. The uptake and sequestration of precious and strategic HMs (e.g., gold, platinum, antimony) through import-storage systems in bioaccumulative GEMs could change the paradigm. Instead of being a bioremediative tool, bioaccumulation could become a bioextractive tool.

### Bioprocess level

Several continuous and batch processes to apply bioaccumulation for bio-HMR have been developed with varying success in lab-scale experiments. While there is potential in using bioaccumulation as a bioextractive tool, lysing HM-saturated cells to purify the concentrated metal implies the biomass is a single-use HMR material, which decreases its economic feasibility when compared to the multi-use conventional technologies. Bioaccumulation needs to be reversible for it to compete with other HMR technologies. Until this obstacle is overcome, it is difficult to envisage how to proceed with the development of bioprocesses to scale-up bioaccumulative GEMs. However, there is value in continuing these developments because the results will inform how “next-generation” bioaccumulative GEMs could be used at industrial scale. This review argues that developments on the cellular level can have the greatest impact on the bioprocess level if *reversible* bioaccumulation is achieved, largely because bioprocess schemes would then need to incorporate an “HM recovery” unit operation to separate HMs from the HM-saturated GEMs for downstream purification and refining.

While the use of GEMs can be contentious due to public concern, technological innovations like those suggested in this review and effective science communication will play major roles in how bioaccumulation research continues. Given rising demands for metal resources and clean freshwater, the development of technologies that can help supply both is crucial. It is this review's hope that by consolidating past efforts in developing bioaccumulative GEMs for bioremediation, future research will begin to explore opportunities to use them for bioextraction, therefore legitimizing bioaccumulation as an industrially feasible bio-HMR technology.

## Author contributions

PD conceived, wrote, and approved the review for publication. RM and AY helped revise the manuscript for submission.

### Conflict of interest statement

The authors declare that the research was conducted in the absence of any commercial or financial relationships that could be construed as a potential conflict of interest.

## Abbreviations

TMS: transmembrane segments
TCDB: Transporter Classification Database
PMF: proton motive force
NTP: nucleoside triphosphate
ABC: ATP-binding cassette
DIGE: differential in gel analysis
IPTG: isopropyl β-D-1-thiogalactopyranoside
PCS: phytochelatin synthase
LC ESI-MS/MS: liquid chromatography electrospray ionization tandem mass spectrometry
ICPL: isotope-coded protein label
MALDI-TOF/TOF MS, matrix assisted laser desorption/ionization: tandem time of flight mass spectrometry
iTRAQ: isobaric tag for relative and absolute quantitation
ICP-AES: inductively-coupled plasma atomic mission spectrometer
ICP-OES: inductively-coupled plasma optical emission spectrometer
ICP-MS: inductively-coupled plasma mass spectrometer
AAS: atomic absorption spectrophotometry.
